# Supplementary material for: Targeting of CXCR4 by the Naturally Occurring CXCR4 Antagonist EPI-X4 in Waldenström’s Macroglobulinemia
Source: Cancers (Basel). 2021 Feb 16;13(4):826. doi: 10.3390/cancers13040826 (PMC7920274; doi:10.3390/cancers13040826)
Supplement: Supplementary file 1 [file cancers-13-00826-s001.zip › cancers-1048040-supp.docx]

**Supplemental Information**

**CXCR4 signaling is counteracted by the naturally occurring CXCR4 antagonist EPI-X 4 in Waldenström’s Macroglobulinemia**

Lisa M. Kaiser^1^, Mirja Harms^2^, Daniel Sauter^2^, Vijay PS Rawat^1^, Mirco Glitscher^3^, Eberhard Hildt^3^, Daniel Tews^4^, Zachary Hunter^5^, Jan Münch^2^, Christian Buske^1,6,*^

**Supplemental Materials and Methods**

*Quantification of CXCR4 Surface Levels by Flow Cytometry*

Cells were stained with anti-CXCR4 antibodies (APC-labeled 12G5 antibody, 555,976 or PE-labeled 1D9 antibody, 551,510 from BD Biosciences, San Jose, CA, USA). Mean fluorescent intensity (MFI) was determined.

*Quantification of CXCR4 protein levels*

For Western blot analysis, BCWM.1 and MWCL-1 proteins were isolated using 100-200 μl ice cold M-PER lysis buffer with Halt™ Protease and Phosphatase Inhibitor Cocktail. Equal amounts of total protein were loaded on a gel and separated using 8 – 16% SDS-PAGE gels, PVDF membranes were blotted using semi-dry Trans-Blot® Turbo™ Transfer System and analyzed using anti-CXCR4 (1:200) monoclonal antibody (Santa Cruz).

*Quantification of phospho and total MAPK*

Activation of ERK1/2 upon stimulation with the ligand CXCL12 (10nM) and the indicated compounds (100µM) was evaluated in BCWM.1 cells by western blot after 10 minutes. The amount of phosphorylated versus total ERK1/2 protein was determined using p44/42 MAPK (Erk1/2) (137F5) Rabbit mAb #4695 Cell signaling 1:1000 and Phospho-p44/42 MAPK (Erk1/2) (Thr202/Tyr204) (E10) Mouse mAb #9106 Cell signaling 1:1000.

**Supplemental Table 1:** Differentially expressed genes between BCWM.1 cells treated with EPI-X4 for 24 h (200 µM) versus inactive peptide. Genes with a FDR < 0,05 and P< 0.05 are listed.

**Supplemental Table 2:** Pathway analysis of differentially expressed and downregulated genes between BCWM.1 cells treated with EPI-X4 for 24 h (200 µM) versus inactive peptide using Enrichr. Genes which are involved in the pathways depicted in Fig.9 B are marked in yellow (adjusted p-value < 0.05).

**Supplemental Table 3:** Pathway analysis of differentially expressed and upregulated genes between BCWM.1 cells treated with EPI-X4 for 24 h (200 µM) versus inactive peptide using Enrichr. Genes which are involved in the pathways depicted in Fig.9 B are marked in yellow (adjusted p-value < 0.05).

.**
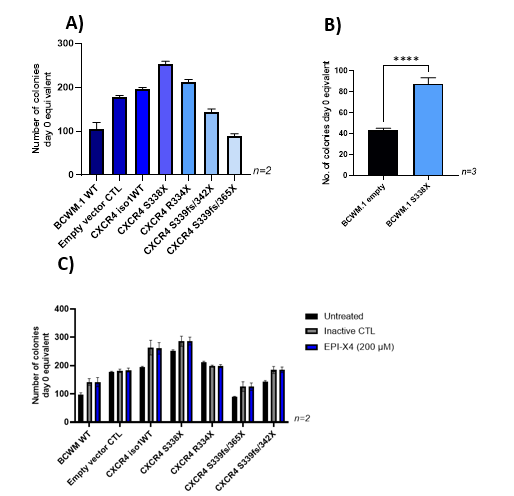
**

**Supplemental Figure 1:** (A) Colony formation ability of BCWM.1 cells transduced with CXCR4 S338X, R334X, S339fs/342X and S339fs/365X as well as the iso1 WT control without CXCL12 stimulation. Mean values (± SEM) of two independent experiments performed in duplicates are shown. (B) Stimulation of BCWM.1 S338X mutant and empty control with 10nM CXCL12. Mean values (± SEM) of three independent experiments performed in duplicates are shown (*** p<0.0001).


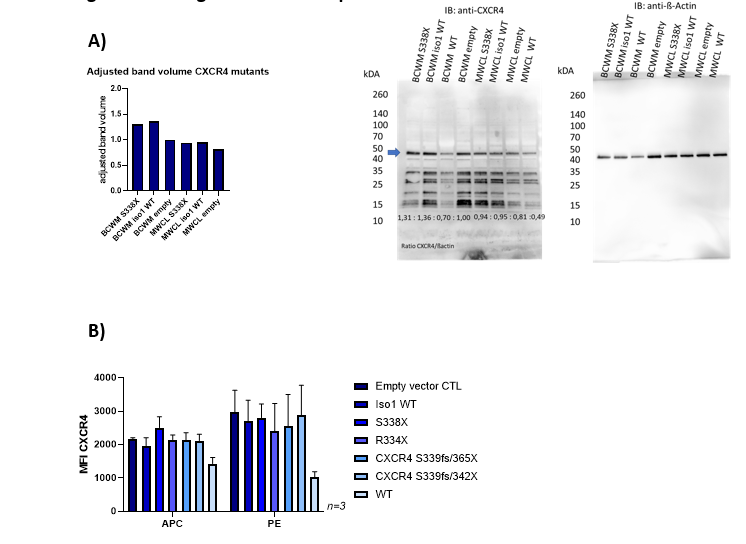


**Supplemental Figure 2:** assessment of endogenous protein expression of the indicated mutants and iso1 WT as well as the non-transduced WT cells by western blot (A) and mean fluorescent intensity of the 12G5 (APC) and 1D9 (PE) CXCR4 epitopes of the BCWM mutants and iso1 WT as well as the non-transduced BCWM WT cells by FACS (B).


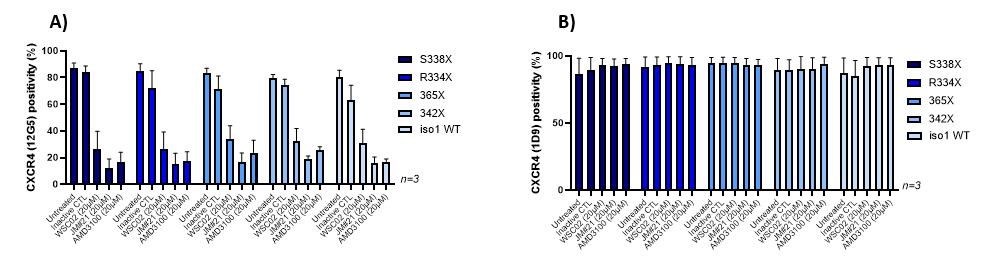


**Supplemental Figure 3:** Successful blockage of all CXCR4 mutants in BCWM.1 cells by low concentrations of optimized derivatives of EPI-X4. BCWM.1 cells were preincubated with 20 μM WSC02, JM#21, AMD3100 or inactive control peptide and then stained with the anti-CXCR4 antibody clones 12G5 (A) or 1D9 (B). While both EPI-X4 derivatives block binding of the anti-CXCR4 clone 12G5 to all CXCR4 variants tested, they do not affect binding of the 1D9 clone. Mean values (± SEM) of one exemplary experiment performed in triplicates are shown.


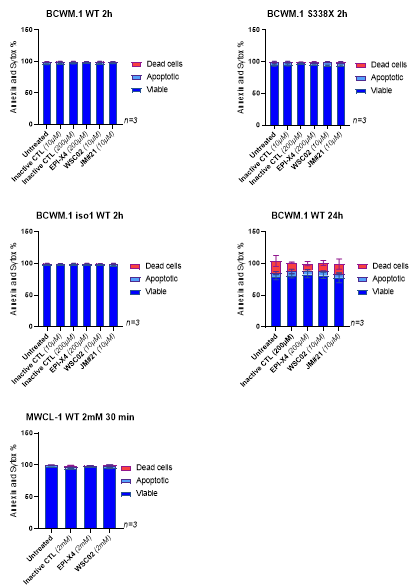


**Supplemental Figure 4:** Annexin staining of the indicated cell lines with different concentrations of the compounds at different timepoints was performed (set-ups are similar to the ones in the experimental conditions eg. migration, incubation for RNA extraction and survival. No significant apoptotic increase was detected compared to the inactive control or the untreated group.

**A)**


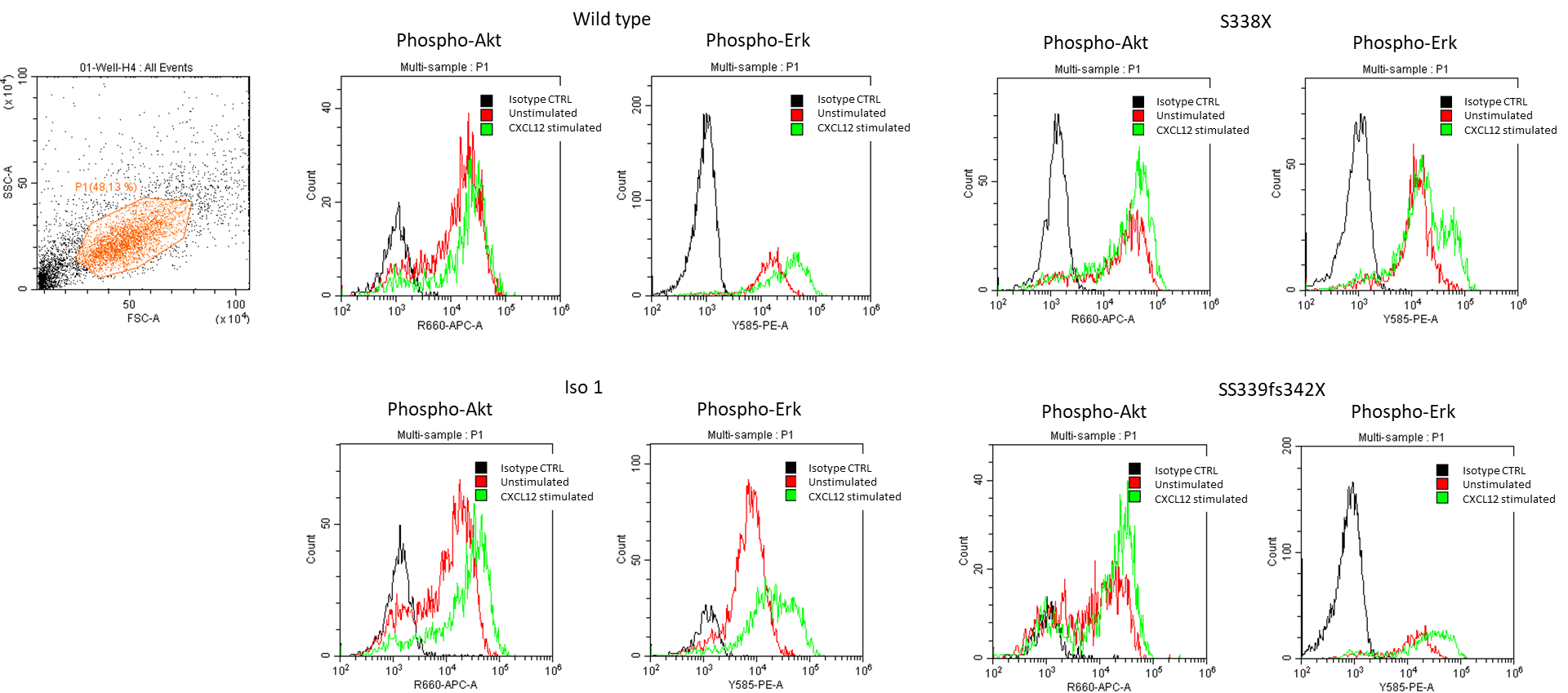


**B)**

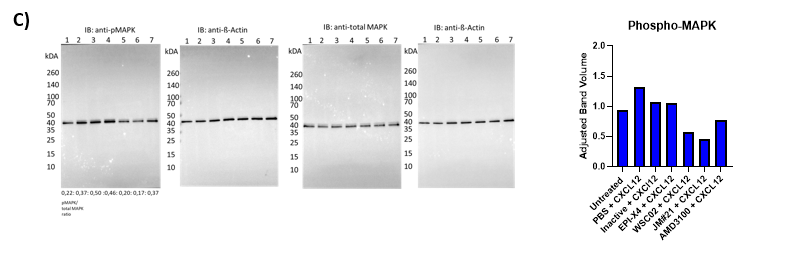


**Supplemental Figure 5:** (A) Representative isotype controls of phoshpho Akt and ERK for wild type cells, iso1WT, S339fs342X and S338X expressing cells unstimulated and stimulated with CXCL12. (B) Facs based assay showing BCWM.1 mutant, ISO1 WT and vector only control cells unstimulated (PBS) or stimulated (CXCL12) without the compounds JM#21 and AMD3100 and with the compounds and the indicated concentrations. Signals normalized by MFI (CXCL12-stimulated cells without inhibitor) / MFI (unstimulated (PBS) cells without inhibitor). Shown are data derived from 2 individual experiments performed in triplicates +/- SD (C) Western Blot analysis of phosphorylated and total MAPK of BCWM.1 WT cells treated with CXCL12 only, inactive peptide, EPI-X4, WSC02 and JM#21 as well as AMD3100 compared to a PBS control without CXCL12 (untreated). 1: untreated, 2: CXCL12, 3: CXCL12+inactive, 4: CXCL12+EPI-X4, 5: CXCL12+WSC02, 6: CXCL2+JM#21, 7: CXCL12+AMD3100.

**
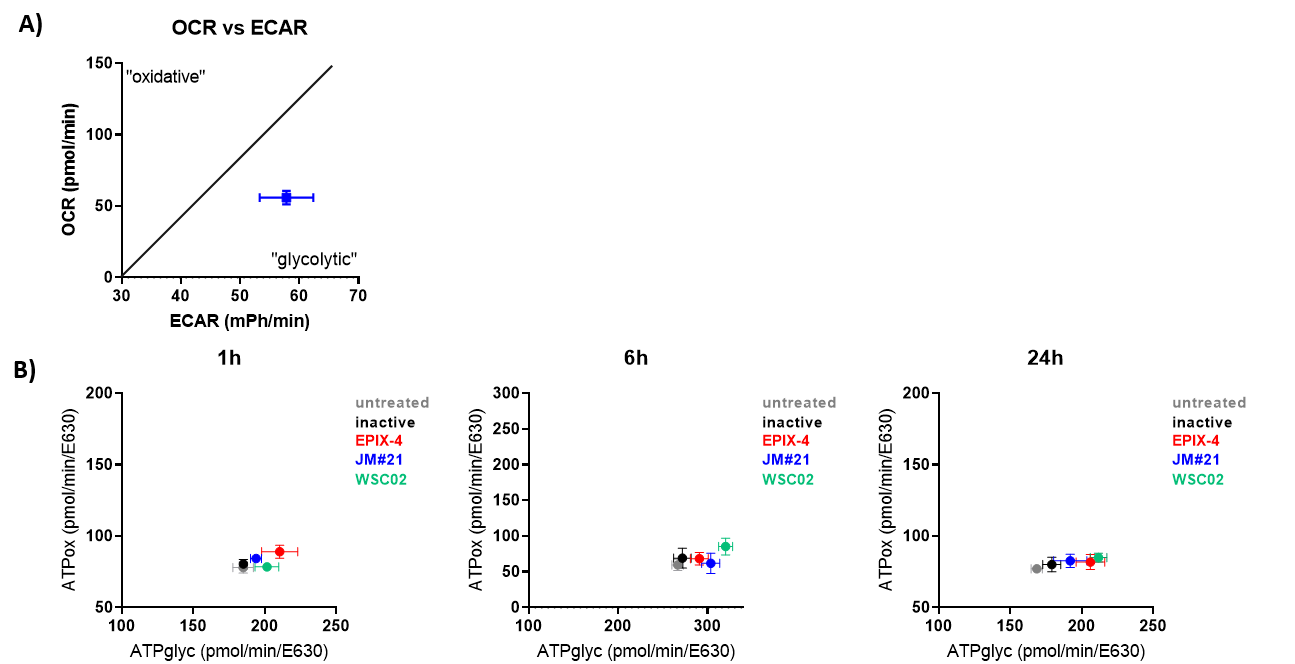
**

**Supplemental Figure 6:** Metabolic assessment using Seahorse XF. (A) BCWM.1 cells were cultivated in normal growth medium (RPMI 10% FBS 1% Penicillin/Streptomycin) and mitochondrial function was assed measuring oxygen consumption rate (OCR) and extracellular acidification rate (ECAR). (B) BCWM.1 cells were cultivated for 1,6 and 24 h under serum-free conditions in the presence or absence of the indicated CXCR4 inhibitors (inactive peptide: 10 µM, EPI-X4: 200µM, WSC02: 10 µM, JM#21: 10µM). Cells were evaluated in terms of their mitochondrial function measuring basal respiration and oligomycin-induced changes in oxygen consumption rate (OCR) and extracellular acidification rate (ECAR), thereby calculating ATP production rates.
